# Pulmonary metastasis of stage I, low-grade endometrioid carcinoma: two case reports and the literature review

**DOI:** 10.3389/fonc.2023.1266485

**Published:** 2023-10-12

**Authors:** Li Wang, Yingxue Li, Lin Han

**Affiliations:** ^1^ Department of Gynecology & Obstetrics, Liaocheng People's Hospital, School of Medicine, Liaocheng University, Liaocheng, China; ^2^ Biomedical Laboratory, School of Medicine, Liaocheng University, Liaocheng, China; ^3^ Department of Pathology, Liaocheng People's Hospital, Liaocheng, China

**Keywords:** pulmonary metastasis, endometrioid carcinoma, early-stage, low-grade, bronchoscope brush liquid-based cytology

## Abstract

Endometrial cancer (EC) is the most common malignant tumor of the female reproductive system, and the majority of ECs are low histological grade and confined to the uterus, resulting in a good prognosis. However, metastasis to the lung from a low-grade and early-stage endometrial endometrioid carcinoma (EEC) is extremely rare. Therefore, it is crucial to accurately differentiate between primary pulmonary malignancy and extra-thoracic malignancy presenting as metastatic disease, and flexible bronchoscopy with tissue acquisition plays a key role in this process. Despite its importance, there is limited literature available on the cytology of metastatic endometrial carcinoma in liquid-based cytology of bronchial brush (BB). In this article, we present two rare cases of lung metastasis from low-grade and early-stage EEC, along with a detailed analysis of the cytologic features observed in BB samples. These cases highlight the significance of cytological and histological pathology, complemented by immunohistochemistry (ICH) analysis, in the diagnosis and management of EEC patients. Pathologists should pay close attention to these aspects, while gynecologists need to be mindful of the follow-up and management of early-stage, low-grade EEC patients. By focusing on these areas, healthcare professionals can effectively contribute to the improved care and outcomes of patients with EEC.

## Introduction

Endometrial cancer is the second most common type of gynecologic cancer and the sixth most common cancer in women globally, with 417,000 new diagnoses made in 2020 (1–4), and the incidence of EC is rising (5, 6). The majority of patients are diagnosed with low-grade, uterine-limited disease (7) and have a good prognosis after undergoing surgical treatment to completely remove the lesions (8–10). The reported 5-year survival rate for endometrial adenocarcinoma is over 89%, with stage I tumors having a rate of 94% (11, 12). Furthermore, the 5-year survival rates corresponding to International Federation of Gynecology and Obstetrics (FIGO) grades 1 and 2 stand at 93% and 94%, respectively (13). However, the recurrence rates of early-stage EC range from 2% to 26% in the literature (14), varying widely among histological subtypes, with rates as low as 7% for patients with low-grade endometrioid adenocarcinoma (15). Histological tumor type is an important prognostic predictor in EC (16).

A retrospective study reported that 2.9% of patients with FIGO (2009) grade 1, non-myometrial invasive tumors without lymphovascular space invasion (LVSI) experienced recurrence (17). It is difficult and critical for risk stratification in early-stage, grade 1 EC (18, 19). A small subset of women with low-grade and early-stage EEC may experience recurrence or distant metastasis (20), such as lung and brain metastases, which are rare occurrences (21). However, the incidence of lung metastasis of cancer patients has been increasing due to improvements in therapeutic options (22) and bronchoscopy evaluation is an important tool for the differentiation between primary lung carcinomas and metastases (23). Therefore, it is crucial to accelerate the study of cytological pathology in these cases for pathologists.

In this article, we present two rare cases of stage I and low-grade endometrioid endometrial cancer with pulmonary metastasis. Furthermore, we provide a detailed description of the bronchoscopy brush liquid-based cytological characteristics of lung metastasis from EEC, which has not been reported previously.

## Case reports

### Case 1

A 70-year-old woman presented with hemoptysis for 6 months and was admitted to our hospital in 2022. Thoracic computed tomography (CT) revealed a mass in the lower lobe of right lung, indicating tumors, as well as multiple small high-density nodules in both lungs.

In 2016, the patient, who had a history of diabetes mellitus (DM) and hypertension, experienced irregular vaginal bleeding for 2 months after menopause. Hysteroscopic biopsy confirmed endometrial cancer, and she subsequently underwent total abdominal hysterectomy with bilateral salpingo-oophorectomy, as well as pelvic and paraaortic lymph node dissection. The diagnosis was endometrioid endometrial carcinoma [FIGO (2009) stage 1A, grade 2]. Adjuvant treatment consisting of six cycles of carboplatin and cyclophosphamide was administered.

### Case 2

A 57-year-old woman presented with a persistent cough of unknown cause for nearly 1 year and was admitted to our hospital in 2022. CT scan revealed multiple small high-density nodules in both lungs.

In 2018, the patient with a BMI of 33 kg/m^2^, was referred to our hospital due to thickened endometrium. Hysteroscopic biopsy confirmed endometrial cancer, and she underwent total abdominal hysterectomy with bilateral salpingo-oophorectomy, as well as pelvic and paraaortic lymph node dissection. The diagnosis was endometrioid endometrial carcinoma [FIGO (2009) stage 1B, grade 1]. Adjuvant treatment included six cycles of carboplatin and cyclophosphamide, as well as pelvic local radiotherapy (DT50Gy/25f).

Both patients underwent flexible bronchoscopy with endobronchial ultrasound (EBUS), which included bronchoalveolar lavage, bronchial brushing, mediastinal lymph node puncture, forceps biopsy, and immunohistochemistry. The results confirmed lung metastasis of endometrial adenocarcinoma.

### Pathology

**Figure 1:**
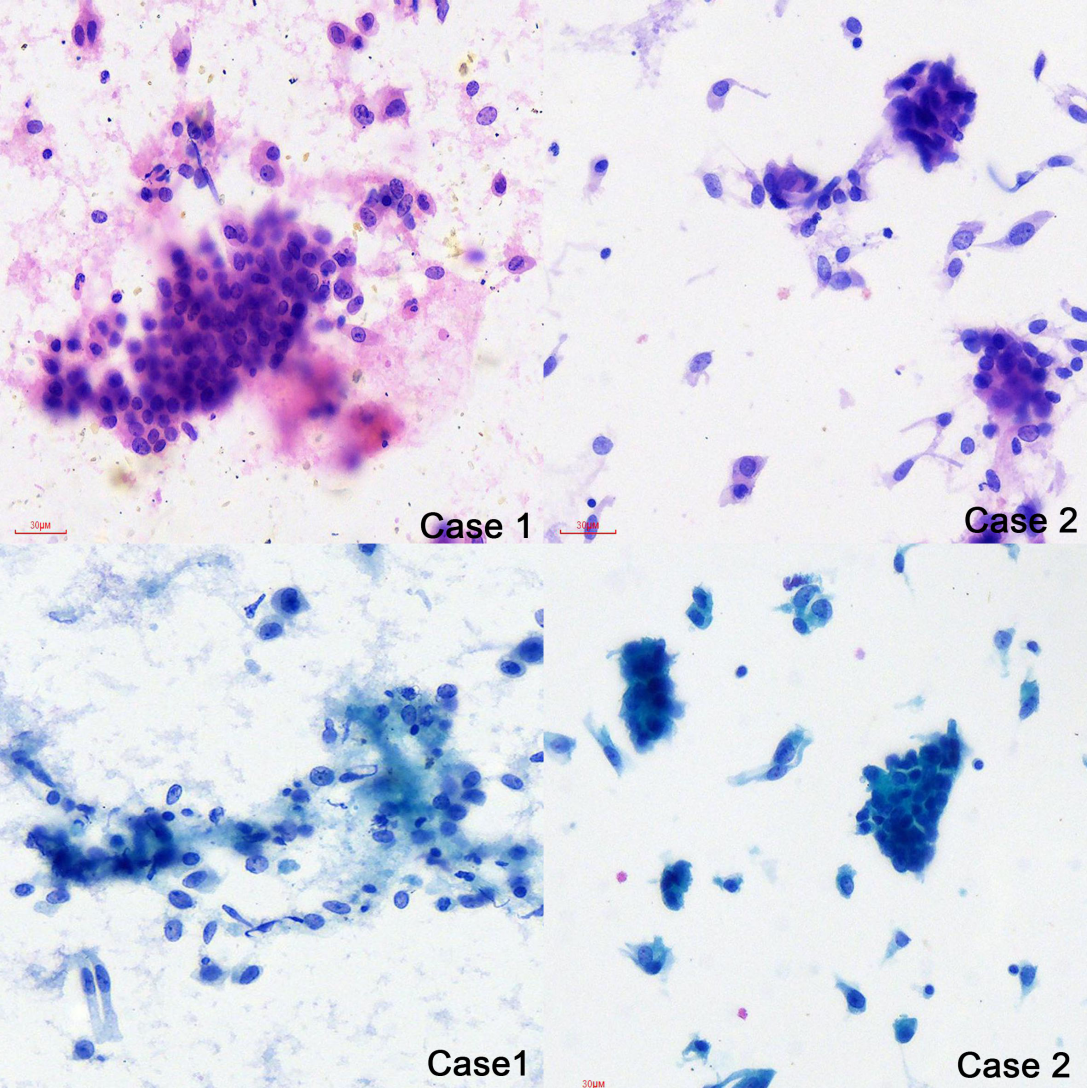
The cytology of bronchial brushing (H&E and Papanicolaou stain ×40).

**Figure 2:**
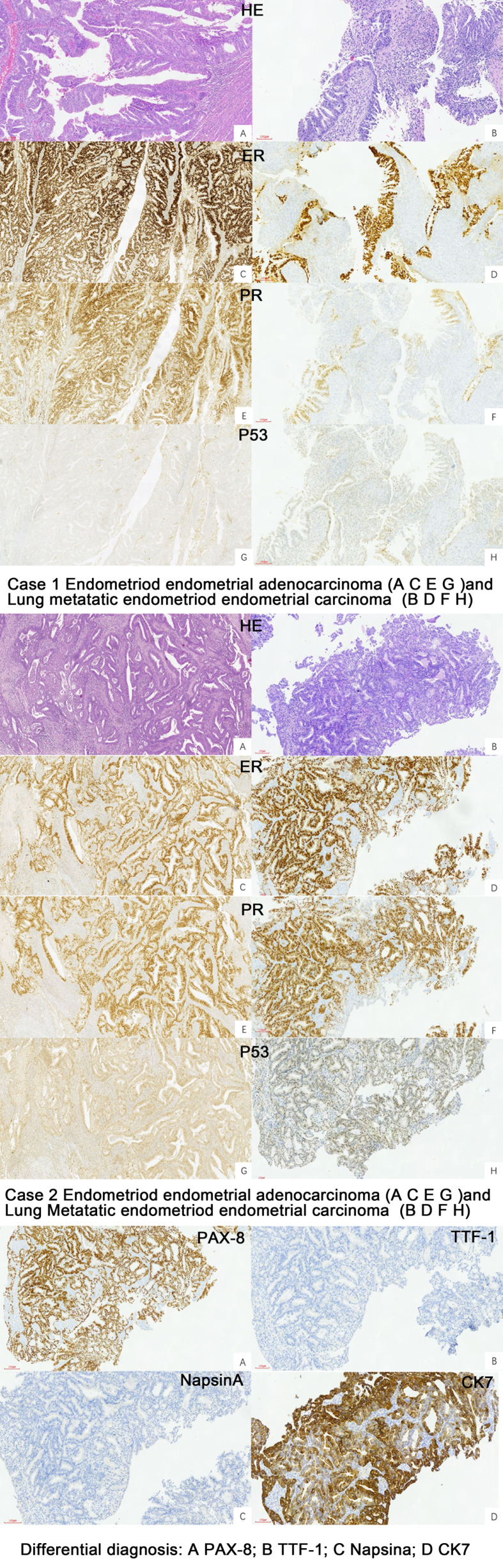
Pathology of the primary EEC and Lung metastatic EEC of the two cases (H&E ×10, IHC stain ×10).

The cytology of bronchial brushing showed cellular morphology that differed from the exfoliated endometrial cancer cells found in cervical fluid-based samples. The morphology was mild, with small atypia, making it difficult to distinguish from bronchial epithelial reactive hyperplasia and carcinoid tumors (**Figure 1**). Subsequent forceps biopsy pathology revealed adenocarcinoma, with one of the two cases showing papillary configurations. Immunohistochemical staining was positive for estrogen, progesterone, wild-type P53, Pax-8, CK7, Ki-67 (60%+), and mottled positive for P16, while negative for TTF-1 and Napsin A (**Figure 2**). The expression of the four MMR proteins (MSH2, MLH1, MSH6, and PMS2) was retained in the metastatic tumor tissues, suggesting microsatellite stable carcinoma.

We reviewed the pathology of the primary endometrial malignancy and found that the histological features were consistent with those of the previous primary EC. Therefore, the nodules were considered metastases from the endometrial malignancy rather than primary lung cancer (**Table 1**).

**Table 1:** Clinical and pathologic information of the two patients.

Case number	Patient age	Primary tumor	Primary pathology examination	First indication of pulmonary metastasis	Bronchoscope brush cytology	Alveolar lavage fluid cytology	Forceps biopsy	Immunohistology	Time interval between primary diagnosis and lung metastasis (years)	FIGO stage (2009)	Re-staged according to 2023 FIGO staging criteria
1	62	Endometrial carcinoma Grade 2, FIGO stage IA	Endometrial carcinoma; Endometrioid cancer; FIGO Grade 2 (Adenoid/cribriform/papillary/solid 10%) Location: Diffused in the cavity Tumor size: 6 × 4.5 × 1 cm Myometrium invasion: <1/2, MELF infiltration was observed; Uterine serous membrane: not involved Para-uterine tissue: None Vaginal: None Cervical invasion: cervical mucosa involved LVSI: less than half of the myometrium with focal LVSI; Adnexal: none; Lymph node status: Pelvic lymph node (−), para-aortic lymph node (−) Immunohistology: wild type P53, ER(high positive, 90%+), PR(high positive, 40%+)	Hemoptysis	Atypical epithelial cells	More histiocytes, ciliated epithelial cells, neutrophils, and a few atypical glandular epithelial cells	Adenocarcinoma, some with filamentous micropapillary structures	ER (high-medium positive, 90%+), PR (medium-week positive, 30%+), wild type P53 +, P16(mottled+), Ki-67(60%+), CK7(+), TTF-1(−), Napsin A(−), Pax-8(+), CK20(−), Villin(−), WT-1(−), Vimentin(−)	6	IVB	IVC
2		Endometrial carcinoma Grade 1, FIGO stage IB	Endometrial carcinoma; Endometrioid cancer; FIGO Grade 1 (Adenoid/cribriform/squamous, with a large number of multinucleated giant cells for special morphology) Location: focal in the cavity Tumor size: 5.5 × 4 × 1 cm Myometrium invasion: >1/2; Uterine serous membrane: not involved Para-uterine tissue: None Vaginal: None Cervical invasion: cervical mucosa involved LVSI: none; Adnexal: none; Lymph node status: Pelvic lymph node (−), para-aortic lymph node (−) Immunohistology: wild-type P53, ER(medium positive, 50%), PR(medium positive, 60%), P16 (mottled+), PAX8(+), Ki67(30%+), CDX2(+), P63(Squamous differentiation+), P40(Squamous differentiation+), Vimentin(+)	Hemoptysis	Atypical epithelial cell mass	Ciliated columnar epithelium, histiocytes, lymphocytes, atypical cells occasionally	Adenocarcinoma	ER (medium–high positive 90%), PR(medium–high positive 90%), wild-type P53 +, P16(mottled+), Ki-67(40%+), CK7(+), TTF-1(−), Napsin A(−), Pax-8(+), MLH1(+), MSH2(+), MSH6(+), PMS2(+)	5	IVB	IVC

### Treatment and follow-up

Both patients were treated with six cycles of combined paclitaxel and carboplatin chemotherapy. The latter patient also received Bevacizumab as part of her treatment. Regular follow-up examinations revealed a significant decrease in tumor size in the lungs after completing chemotherapy.

## Discussion

Prognosis for patients with endometrial adenocarcinoma is generally good. However, disease recurrence, either local or distant, occurs in 7% to 15% of diagnosed patients (15), particularly those with endometrial endometrioid carcinoma (EEC) and those with non-endometrioid histology (24–26). Although 5% to 10% of low-grade EEC patients will experience either local recurrence or distant metastasis (17, 27, 28), the occurrence of distant organ metastasis is still very rare (29) for the stage I with low-grade EEC. In the domain of endometrial carcinoma, the histological classification embodies a seminal prognostic determinant. The two case instances under our scrutiny, which now stand subjected to the nuances of the revised 2023 FIGO staging, find their classification within the ambit of non-aggressive histological phenotypes. This realm encapsulates the realm of low-grade entities, constituting Grades 1 and 2 endometrioid endometrial carcinomas, while the antithetical cadre of aggressive histological counterparts encompasses the expanse of Grade 3 EECs, serous carcinoma (SC), clear cell carcinoma (CCC), mixed carcinoma (MC), undifferentiated carcinoma (UC), carcinosarcoma (CS), and mesonephric-like and gastrointestinal type mucinous carcinomas (16). Molecular features can be used to estimate risk of recurrence and hence survival (30–33). The Cancer Genome Atlas (TCGA) has meticulously categorized endometrial carcinomas into four distinct classifications (16, 34): (1) POLE/Ultramutated: This category is characterized by the presence of somatic inactivating hotspot mutations within the POLE exonuclease domain, resulting in an exceptionally elevated mutational burden. Irrespective of the histological grade, tumors displaying POLE mutations exhibit a remarkably favorable prognosis. (2) Microsatellite Instability-High/Hypermutated: This subset entails endometrioid endometrial carcinomas (EECs) or undifferentiated carcinomas that exhibit a deficiency in mismatch repair (MMRd) leading to microsatellite instability. This category bears an intermediate prognosis. (3) Somatic Copy-Number Alteration High/Serous-Like (SCNA-High): Marked by a low mutation rate, nearly ubiquitous TP53 mutations (95% prevalence), and an exceedingly unfavorable prognosis. While the majority of these malignancies manifest as serous carcinomas, a fraction of up to 25% comprises endometrioid carcinomas (primarily high-grade) and carcinosarcomas. (4) Somatic Copy-Number Alteration Low (SCNA-Low): This class encompasses endometrioid endometrial carcinomas and clear cell carcinomas characterized by scant copy-number alterations and a diminished mutational burden. In this study, we present two cases of low-grade (grades 1 and 2) and early-stage (stage IA and IB) endometrial adenocarcinoma (positive ER/positive PR/wild-type p53) with distant metastasis. If available and feasible, it is recommended to do molecular classification testing (POLEmut, MMRd, non-specific molecular profile [NSMP], and p53 abnormal [p53abn]) in all patients with endometrial cancer to enable the meticulous stratification of patients into discrete prognostic risk groups and to furnish invaluable insights that possess the potential to exert influence over determinations concerning adjuvant and systemic therapeutic strategies (16). The FIGO (2023) staging assign stages I and II based on meticulous surgical–anatomical and histological assessments. Within the realm of molecular classification, the statuses of POLE mutation and p53abn have emerged as significant indicators. p53abn status has been indicative of an unfavorable prognosis; however, in the context of our two cases, it is noteworthy that all instances exhibited wild-type p53 status. The interval from primary diagnosis to metastasis was 6 years and 4 years, respectively. A previous study reported a mean interval time of 4.9 years based on the analysis of 8 endometrial carcinoma patients without subtyping of grade or stage (35).

Pulmonary metastasis is a common occurrence in cases of metastatic carcinoma. In relation to gynecologic tumors, the lungs are reported to be the most frequent site of metastasis from low-grade endometrial stromal sarcoma (36–38), although this is uncommon (35). However, recent reports indicate that lung metastasis is the most common distant organ metastasis in endometrial tumors (39–41), with carcinosarcoma having a significantly higher rate of lung metastasis compared to other histological types, and undifferentiated tumors had the highest rate of lung metastasis when considering tumor grade (29). A study reported that stage IA endometrial adenocarcinoma can exhibit distant metastasis, often spreading hematogenously to the lungs. However, in that study, the metastasis occurred in cases with the papillary serous histological subtype (42). Endometrial mesonephric-like carcinomas (MLCa), constituting an approximate fraction of 1% among endometrial carcinomas (43), have a high incidence of lung metastasis (44); one study has documented that MLCa frequently undergoes recurrence accompanied by distant metastases, with the pulmonary locale predominating (comprising 64% of metastatic cases) (45). MLCa also exhibit a diverse spectrum of morphological presentations, often resulting in their inadvertent under-identification or misclassification as low-grade (grade 1 or 2) endometrioid endometrial carcinomas (46, 47). Immunohistochemistry could help to differentiate the diagnosis, and the MLCa components were characterized by the variable expression of markers supportive of mesonephric differentiation (GATA3, TTF1 and CD10) and lack of hormone receptor (ER and PR) expression, whereas EEC shows the opposite staining pattern (48) when performed. The two patients we reported both had stage I endometrial endometrioid carcinoma (ER and PR positive).

The risk of endometrial cancer increases with age and BMI (34); the first case in our report involved a 70-year-old patient with a history of DM, while the second case involved a 57-year-old patient with a BMI of 33 kg/m^2^. Evidence from a previous report indicated that DM is a poor prognostic factor in patients with low-grade EEC, specifically those with KRAS mutation (49). BMI and increases with age are the main risks of endometrial cancer; it has the strongest link to obesity among the 20 most common types of tumors. Each 5 kg/m^2^ increase in BMI is associated with a 54% higher risk of cancer (50, 51). Furthermore, obesity is considered a risk factor for recurrence (52). Obesity creates a proinflammatory environment characterized by high levels of circulating interleukin-6, tumor necrosis factor-α, C-reactive protein, and a relative deficiency of protective immune cell types (53, 54). Obesity also leads to a hyper-estrogenic state due to the peripheral aromatization of adrenal androgens to estrogen by adipose tissue (55). The prevailing theory regarding endometrial carcinogenesis suggests that natural progesterone deficiency contributes to an unopposed estrogen excess driven by obesity in postmenopausal women (56). Therefore, weight management should be included as an essential component of follow-up care for patients with EEC. Optimizing survivorship through weight loss and lifestyle interventions could improve both the survival and quality of life for individuals with endometrial cancer (57).

Imaging techniques play a crucial role in distinguishing between primary and metastatic tumors in the lungs. CT scans are the standard imaging modality for assessing the extent of the disease (58). However, clinical presentation and radiographic findings may exhibit significant overlap between lung metastases and primary lung cancer. To further differentiate these conditions in chest imaging, bronchoscopy is commonly employed as a diagnostic tool for lung cancer (59). Flexible bronchoscopy, which includes procedures like bronchial brushing and forceps biopsy, is particularly useful in evaluating peripheral lung abnormalities. In our report, both patients underwent flexible bronchoscopy with endobronchial ultrasound (EBUS) to obtain accurate diagnoses. This involved techniques such as bronchoalveolar lavage, bronchial brushing, mediastinal lymph node puncture, and forceps biopsy. These procedures allowed for the collection of small histological or cytological samples, enabling tissue biopsies and brushings to contribute to an accurate diagnosis.

Cytological and histological examination, along with immunohistochemistry, are crucial for diagnosing lung metastases. However, there is limited information available regarding the characteristics of liquid-based cytology using bronchial brush samples. In our study, we described two cases involving the cytology of bronchial brushes. The cell morphology observed was distinct from the exfoliated endometrial cancer cells found in cervical fluid-based samples. In these cases, the cellular changes were mild, displaying small atypia, which made it challenging to differentiate them from bronchial epithelial reactive hyperplasia and carcinoid tumors. Following the cytology report, forceps biopsy was performed, and the pathology revealed adenocarcinoma, with one of the cases demonstrating papillary configurations. The utilization of IHC has significantly enhanced the precision of diagnostic categorization in the realm of lung carcinomas, and the immunoprofiles of adenocarcinomas arising from the female genital tract (cervix, endometrium, fallopian tube, and ovary) differ depending on the tumor histotype and primary sites (33). Immunohistochemical analysis in our cases showed positive staining for wild-type p53, positive estrogen receptor (ER), progesterone receptor (PR), Pax-8, CK7, and Ki-67 (60%+). Additionally, there was patchy positive staining for P16 that could help us to distinguish it from SC and EEC, while staining was negative for TTF-1 and Napsin A. The manifestation of wild-type p53 was discerned in our cases, a finding harmoniously aligned with the primary endometrioid endometrial carcinoma immunohistochemical staining. This congruence in p53 status not only bolsters the diagnostic correlation but also plays a pivotal role in guiding the selection of appropriate adjuvant therapeutic modalities. Positive for ER and PR not only helps us to compare the pathology with primary EEC, but also furnishes a decisive means for differentiation from primary lung carcinoma or endometrial MLCa. Pax-8 exhibits a remarkable capacity for recognizing the majority of adenocarcinomas within the female genital tract, a characteristic that starkly contrasts with its applicability to lung adenocarcinomas (60, 61); the positive Pax-8 immunostaining observed in our cases has significantly contributed to the precise elucidation of the diagnosis, firmly establishing the origin of the lung metastasis as stemming from EEC. TTF1 staining is a critical single marker for adenocarcinoma in lung cancer, with Napsin A also showing some diagnostic utility as a secondary marker for adenocarcinoma in lung tumors (62); a combination of TTF1 and Napsin A may yield greater sensitivity for lung adenocarcinoma (63), and the absence of both TTF1 and Napsin A immunoreactivity played a pivotal role in solidifying the diagnosis for the cases. To further validate the findings, we reviewed the pathology results of the primary endometrial malignancy, which exhibited a similar pattern to the lung lesions.

**Figure 3:**
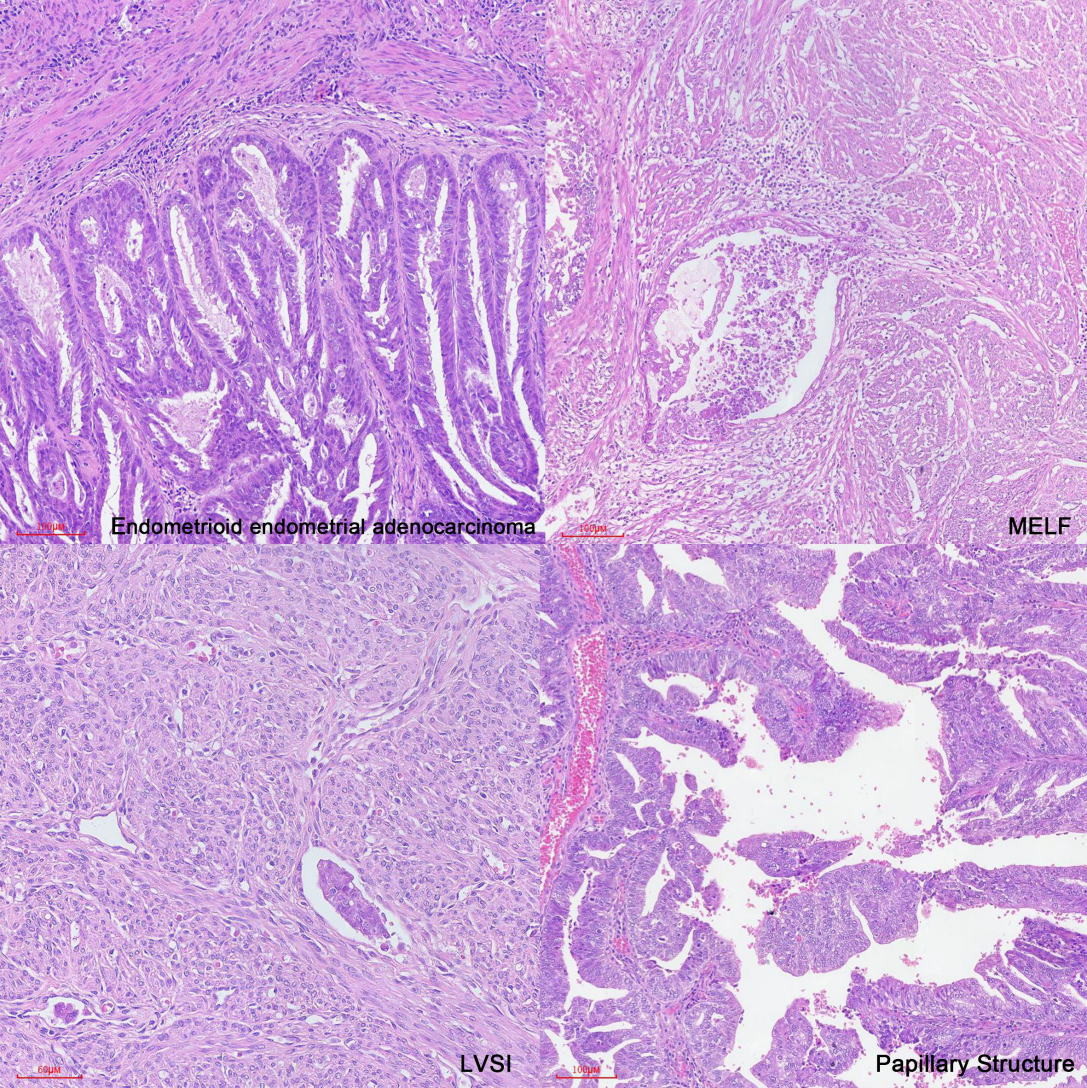
Primary pathological structure of EEC, MELF, LVSI and Papillary Structure (H&E ×10).

In case 1, the patient had a low prognostic risk (34) as she was stage IA low-grade (grade 2) endometrioid carcinoma and focal LVSI. Classic features associated with distant recurrence included age, stage at presentation, deep myometrial invasion, and LVSI (11). However, we also observed the presence of microcystic, elongated, and fragmented (MELF) pattern of myoinvasion (**Figure 3**), which is considered a significant feature of recurrence or distant metastasis. MELF patterns are newly described patterns that are typically associated with FIGO grade 1 or 2 endometrioid adenocarcinoma. These patterns could potentially indicate epithelial mesenchymal transition in carcinomas, facilitating infiltration into the surrounding stroma and promoting tumor progression (64, 65). LVSI, characterized by the presence of tumor emboli within lymphatic, capillary, or venous channels (66, 67), is associated with an increased likelihood of metastasis to lymph nodes and other sites (68).

For the patient in case 2, she had intermediate risk as she was stage IB endometrioid carcinoma and low-grade (grade 1) with negative LVSI (34). The immunohistochemistry staining of the primary tumor revealed medium positive expression of ER (50%) and PR (60%). According to a recent study, decreased expression of ER/PR detected by immunohistochemistry can serve as a valuable prognostic biomarker for identifying low-grade EEC that may have distant metastasis (49).

To the best of our knowledge, there have been several reported cases in the current literature of endometrial carcinoma with lung metastases. However, scant literature exists elucidating pulmonary metastases of stage I low-grade (grades 1 and 2) endometrioid endometrial carcinomas, an exceedingly rare phenomenon. In our analysis, we examined the high-risk factors in terms of clinical features and histological pathology, emphasizing the necessity of precise management for early-stage EEC patients, particularly those with a history of DM or obesity. Additionally, we highlighted the importance of paying closer attention to patients exhibiting LVSI, MELF patterns, and a decrease in ER and PR expression.

## Conclusion

The occurrence of pulmonary metastases in stage I, low-grade (1 and 2) EEC is extremely rare. Therefore, gynecologists should pay close attention to the management and follow-up of early-stage, low-grade EEC patients. It is crucial for cytological pathologists to recognize the characteristics of bronchoscopy brush liquid-based cytology in cases of lung metastasis from EEC. Early and accurate diagnosis of metastatic EEC is important, as it allows for appropriate treatment to be administered to these patients.

## Data availability statement

The raw data supporting the conclusions of this article will be made available by the authors, without undue reservation.

## Ethics statement

Written informed consent was obtained from the individual(s) for the publication of any potentially identifiable images or data included in this article.

## Author contributions

LW: Writing – review & editing, Project administration, Supervision, Writing – original draft. YL: Writing – review & editing, Methodology. LH: Methodology, Writing – review & editing, Investigation.
